# The learning curve of novel implant total knee arthroplasty system in high-volume university center

**DOI:** 10.1051/sicotj/2025041

**Published:** 2025-08-07

**Authors:** Simon Messe, Guillaume Mesnard, Hannes Vermue, Enrico Festa, Elvire Servien, Anthony Viste, Cécile Batailler, Sébastien Lustig

**Affiliations:** 1 Univ Lyon, Claude Bernard Lyon 1 University, IFSTTAR, LBMC UMR_T9406 43 boulevard du 11 Novembre 1918 69622 Villeurbanne Cedex France; 2 Orthopaedics Surgery and Sports Medicine Department, FIFA Medical Center of Excellence, Croix-Rousse Hospital, Lyon University Hospital 103 Grande Rue de la Croix Rousse 69004 Lyon France; 3 Department of Orthopaedic Surgery, Ghent University Hospital C. Heymanslaan 10 9000 Gent Belgium; 4 Department of Public Health, Orthopedic Unit, “Federico II” University Via Pansini 5 80131 Naples Italy; 5 LIBM – EA 7424, Interuniversity Laboratory of Biology of Mobility, Claude Bernard Lyon 1 University 27-29 Boulevard du 11 Novembre 1918 69100 Villeurbanne France; 6 Hospices Civils de Lyon, Hôpital Lyon Sud, Chirurgie Orthopédique et Traumatologique 165 Chemin du Grand Revoyet 69495 Pierre Bénite France; 7 University Lyon, Claude Bernard Lyon 1 University, Gustave Eiffel University, IFSTTAR, LBMC UMR_T9406 25 Avenue François Mitterrand 69675 Bron Cedex France

**Keywords:** Total knee arthroplasty, Learning curve, CUSUM analysis, Operative time, Postoperative complications, Surgical performance

## Abstract

*Introduction*: The learning curve associated with adopting new surgical systems in total knee arthroplasty (TKA) can significantly impact surgical efficiency and patient outcomes. This study aimed to evaluate the evolution of operative time with the KNEO^®^ (Groupe Lépine, Genay, France) posterior stabilized knee system and to analyze the learning curve for postoperative complications to achieve surgical proficiency. *Method*: This retrospective, multicentric study analyzed 481 patients who underwent primary TKA with the KNEO^®^ implant in a high-volume university center between 2020 and 2024. The evolution of operative time and postoperative complications requiring reoperation surgery were evaluated, with a follow-up period extending until January 2025, during which complications were monitored. The study included 481 patients with a mean age of 71.7 ± 8.0 years and a mean Body Mass Index of 29.0 ± 4.0 kg/m^2^. The cohort comprised 308 female (64%) and 173 male (36%) patients. *Results*: The mean operative time significantly decreased from 83.5 min in the initial case to 63.0 min after 481 cases (*p* < 0.001). The learning curve showed an initial learning phase with high variability, followed by stabilization around 150 procedures and subsequent optimization. Postoperative complication rates showed a 31.9% reduction per group of 100 patients (β = −0.3848, *p* = 0.0075), indicating improved surgical proficiency and patient safety over time. *Conclusion*: The findings suggest that the KNEO^®^ system follows a measurable learning curve, with operative efficiency and complication rates improving as case volume increases. These results emphasize the importance of structured training and experience accumulation in optimizing patient outcomes when implementing new implant technologies.

## Introduction

Adopting new techniques or technologies in knee arthroplasty presents inherent challenges for surgeons, often requiring a period of adaptation and skill acquisition [1]. The process of mastering a new surgical system involves a learning curve, which can be defined as the improvement in surgical proficiency and consistency of outcomes over time, as the surgeon gains experience with a specific technique or technology [2, 3]. This curve varies significantly between different techniques and is influenced by multiple factors, including surgeon expertise, the surgical team, case volume, patient turnover, and the healthcare setting [4]. Understanding the duration, extent, and variability of the learning curve is crucial, as it can directly impact surgical outcomes, including complication rates and operative times.

Shorter operative times, for instance, have been associated with lower infection risks, reduced anesthesia exposure, less physiological stress for the patient, and overall improved cost-effectiveness [5]. These improvements in surgical efficiency and safety are especially relevant in the context of newer surgical systems, which may necessitate a steeper initial learning curve before surgeons can achieve optimal outcomes.

For example, recent studies have examined the learning curve for minimally invasive techniques in total knee arthroplasty (TKA), highlighting the need for a significant number of procedures before surgical times stabilize and complication rates diminish [2, 3]. Additionally, new implant designs, such as the KNEO^®^ Knee System (Groupe Lépine, Genay, France), a posterior stabilized implant launched in 2020, have been shown to improve alignment accuracy, which could further reduce complications and enhance outcomes [6, 7].

The surgical learning curve can be quantitatively assessed using methods like the cumulative sum control chart (CUSUM), which detects shifts from the learning phase to the post-learning phase by identifying inflection points in surgical performance data [8]. This method has been successfully applied to assess learning curves in various surgical fields, including TKA, to evaluate the effect of experience on outcomes.

This study investigates the learning curve for the KNEO^®^ total knee arthroplasty performed at two high-volume university centers. The primary aim is to assess the relationship between the number of procedures and surgical duration, with the hypothesis that fewer than 100 procedures will be required to overcome the learning curve. A secondary aim is to evaluate the learning curve for surgical post operative complications, with a focus on how complication rates decrease as experience with the system increases.

## Method

### Study design

This was a retrospective study that included all patients who underwent primary total knee arthroplasty using the Posterior Stabilized KNEO^®^ (Groupe Lépine, Genay, France) implant at one tertiary referral centers specialized in primary and revision knee arthroplasty between January 2020 and May 2024. This study was approved by the national ethical committee (approval number [ID-RCB 2020-A00414-35]).

### Setting and participants

**Figure 1:**
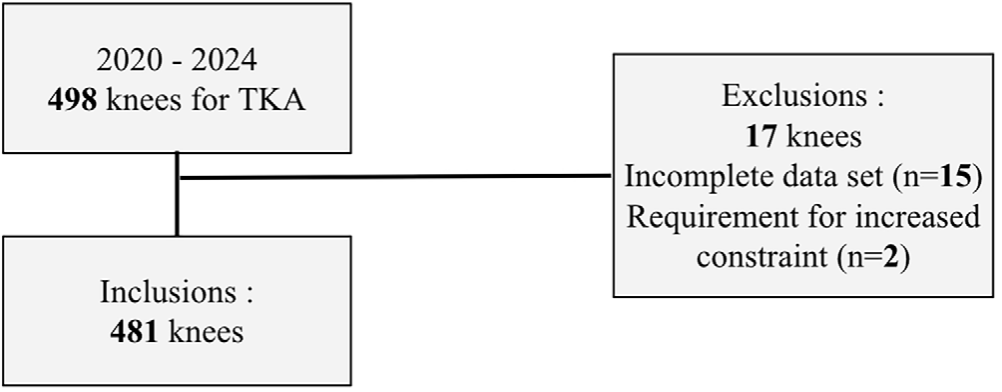
Patient selection flowchart.

The study included patients requiring TKA for diagnoses of primary osteoarthritis, inflammatory arthritis, avascular necrosis, or post-traumatic arthritis. Patients with incomplete data were excluded from the analysis (Figure 1). In addition, two patients were excluded because intraoperative events required a change in the prosthetic constraint level. Specifically, both patients sustained a medial collateral ligament (MCL) rupture during surgery, which necessitated conversion from a posterior-stabilized implant to a more constrained prosthetic design.

The patient demographics are summarized in Table 1. A total of 498 patients were eligible for review from June 2020 to May 2024. The study included 481 patients.

**Table 1:** Baseline demographics. BMI = Body Mass Index, W = Women, L = Left, ASA = American Society of Anesthesiologists.

Variable	Value	Range
Mean age, years (SD)	71.7 (8.1)	35–91
Mean BMI, kg/m^2^ (SD)	29.0 (5.2)	15–55
Sex, female % (*n*)	64 (308)	
Laterality, left % (*n*)	42.8 (206)	
Mean surgical time, minutes (SD)	73.5 (20.20)	
ASA 1 % (*n*)	6.9 (33)	
ASA 2 % (*n*)	65.5 (315)	
ASA 3 % (*n*)	27.3 (131)	
ASA 4 % (*n*)	0.4 (2)	
Primary osteoarthritis % (*n*)	88.8 (427)	
Post-traumatic osteoarthritis % (*n*)	7.7 (37)	
Post-necrotic osteoarthritis % (*n*)	1.4 (7)	
Post-rheumatic osteoarthritis % (*n*)	2.0 (10)	

### Surgical technique

All patients underwent a standardized enhanced recovery protocol, including spinal anesthesia, early mobilization, appropriate pain management, and thromboprophylaxis as per national guidelines [9]. The procedures were performed through a subvastus, medial parapatellar or lateral parapatellar surgical approach, using a manual measured resection technique. Intramedullary referencing was utilized for both the femur and tibia. All TKAs were aligned according the restricted kinematic alignment philosophy has previously been described by Blakeney and Vendittoli [10]. The femoral and tibial component were always cemented. Patellar resurfacing was performed selectively based on the degree of patellar osteoarthritis observed during the surgery.

The surgeries were performed by multiple surgeons from the department, including 7 senior surgeons and 10 junior surgeons. The individual learning curves of the surgeons were not separately analyzed.

### Data collection

Routine departmental follow-up occurred at two months, one year, and every 5 years thereafter. The clinical data collected from the electronical patient files included the patient’s ASA score, Body Mass Index (BMI), range of motion, and International Knee Society Score (IKS), and operative time in minutes, measured from skin incision to wound closure.

Postoperative complications were defined as any adverse events that required additional surgical treatment.

### Statistical analysis

The cumulative summation sequential analysis tool (CUSUM) [11] was used to assess the learning curves in TKA for operative time and complication rates.

For continuous variables CUSUM works by continuously summing the differences between an observed CUSUM works by continuously summing the differences between consecutively observed values (e.g., operative time) and a predefined target of 73.5 min, which represents the mean operative time for the cohort. A stable curve suggests consistent performance. A rising curve indicates an increase in variability or prolonged surgical times. A falling curve reflects improved efficiency. In case of a binary variable, such as whether or not a complication occurred, a Bernoulli CUSUM analysis was used with a reference proportion of 6%.

To analyze the variation in complication rates across patient groups, we employed a Poisson regression model within a generalized linear modeling framework.

The model was fitted using a Poisson distribution with a log link function to account for the count-based nature of the data. To assess the significance of the patient group as a predictor, we conducted a Wald test, considering a *p*-value < 0.05 as the threshold for statistical significance. Model fit was evaluated using deviance residuals and the Pearson chi-square statistic to determine the adequacy of the model in explaining the observed variance.

Statistical analyses were performed using SPSS (version 26.0, IBM, NY, US) and R (version 4.0.5, R Foundation, Vienna, Austria).

## Result

### Evolution of surgical time and learning curve

The analysis of the learning curve revealed a progressive reduction in surgical time over time. The initial mean operative time was 83.5 min, with a regression slope of −0.039 (*p* < 0.05), indicating a statistically significant decrease in operative duration.

**Figure 2:**
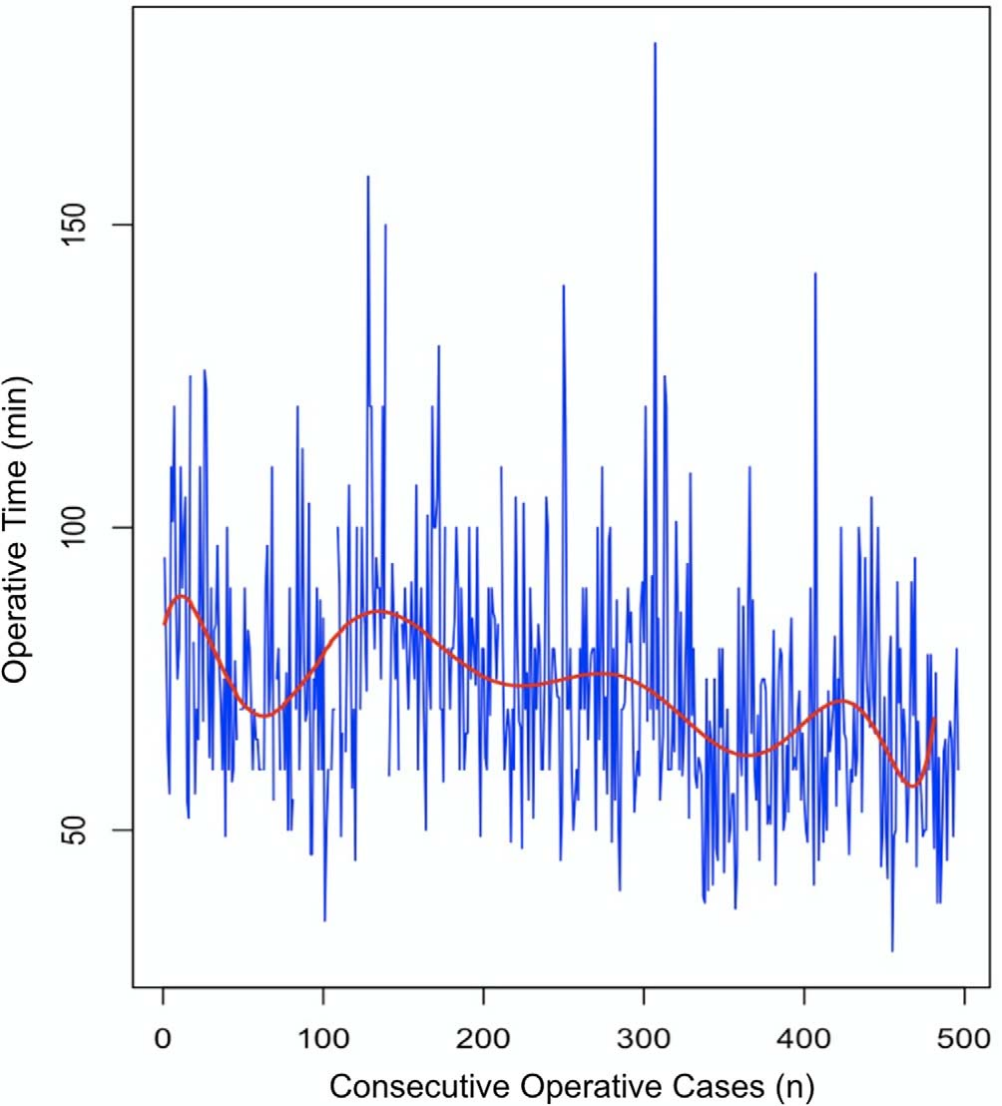
Trends in operative time over 481 consecutive total knee arthroplasty cases performed with the KNEO^®^ system. The blue lines represent individual operative times (in minutes) for each consecutive case. A red LOESS smoothing curve highlights a progressive downward trend.

The chronological analysis of operative times (Figure 2) demonstrated a total reduction of 20 min, representing 16.7% decrease compared to the initial operative time. By the end of the observation period (*t* = 481), the mean surgical duration had decreased to 63 min, confirming the effect of surgical learning.

Linear regression applied to the data showed a significant negative slope, indicating that the operative time decreased by 4 min per 100 procedures on average. The associated *p*-value was <0.05, confirming the statistical significance of this trend.

### CUSUM analysis on surgical time

**Figure 3:**
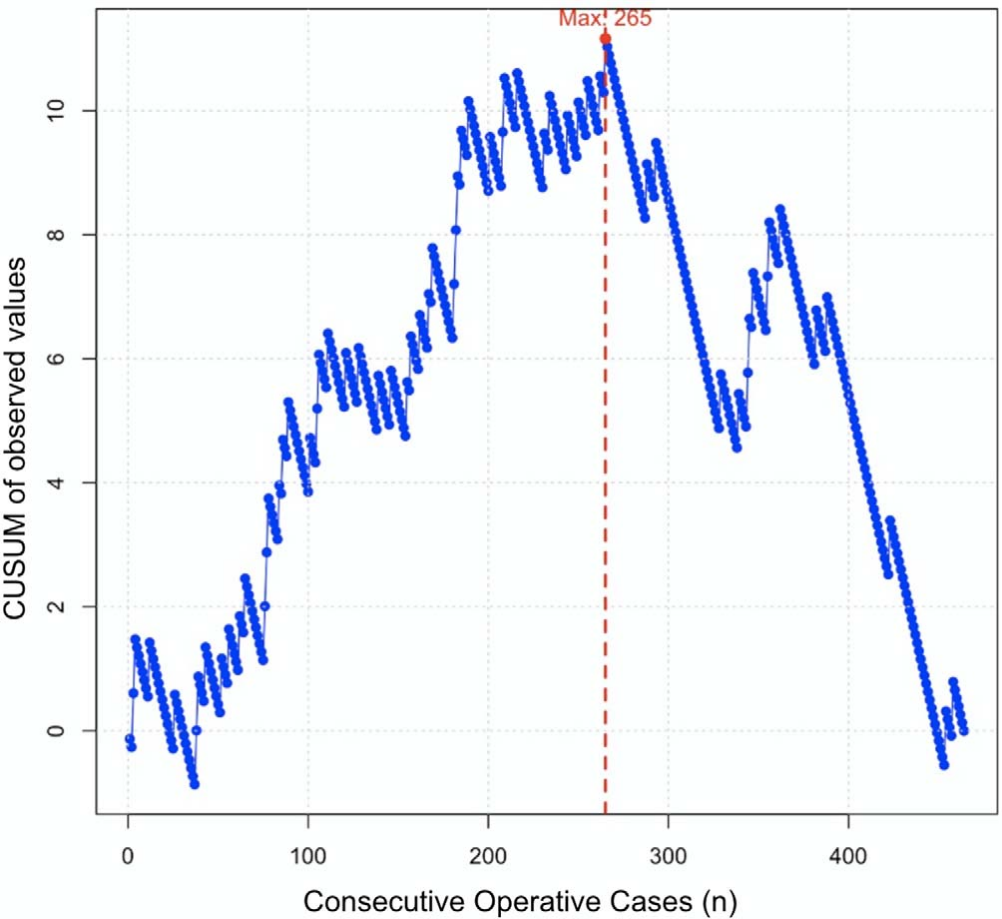
CUSUM Analysis of Operative Time. The blue curve represents the cumulative deviation of each case’s operative time from the overall mean. The trajectory reveals three distinct phases: an initial learning phase with a steady rise in CUSUM values; a stabilization phase with a plateau around case 265; and a subsequent optimization phase characterized by a marked decline in CUSUM values. The red dotted line indicates the inflection point at case 265, marking the transition beyond the learning phase.

The CUSUM curve on surgical time (Figure 3) identified three phases in the evolution of operative duration. During the initial learning phase, spanning the first 150 cases, a rapid increase in CUSUM values was observed, reflecting longer and more variable operative times characteristic of the learning process. Around 150 cases, the stabilization phase began, marked by more consistent operative times, indicating progressive mastery of the technique. Beyond 316 cases, the optimization phase was reached, with a clear decrease in CUSUM values, suggesting refined surgical techniques and improved efficiency. Despite this overall stabilization, occasional peaks in operative time were noted.

### CUSUM analysis of complications requiring reoperation surgery

**Figure 4:**
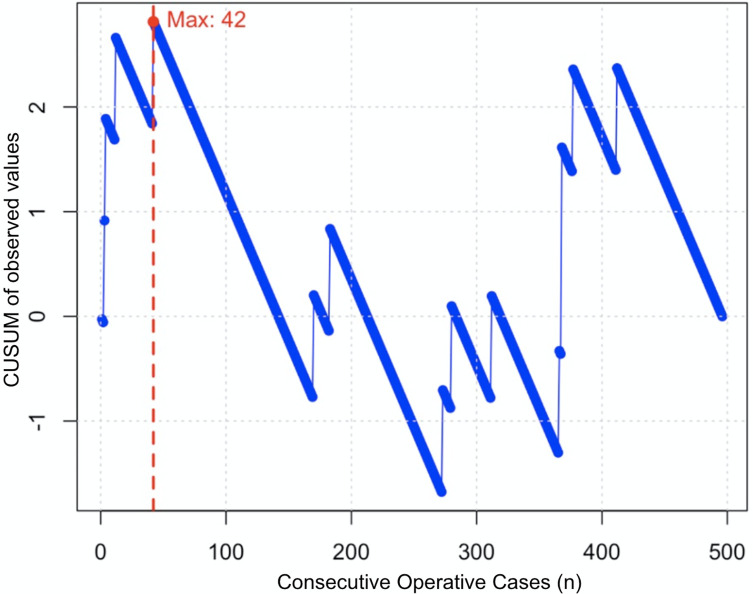
CUSUM analysis of postoperative complications. Each point represents a binary outcome (presence or absence of complication), with deviations from a predefined reference value (6%) cumulatively summed. The initial rising curve up to case 42 reflects a higher-than-expected complication rate during the learning phase. The inflection point at case 42 (red dot and dashed line) marks the transition to improved surgical performance. After this point, the consistent downward trend indicates reduced complication rates.

The CUSUM curve (Figure 4) shows an initial increase up to 42 TKA, indicating a learning phase characterized by a higher complication rate, with a higher complication rate affecting 6% of patients before case 42. After this point, the curve declines, reflecting an improvement in outcomes, followed by a stabilization phase towards the end of the observation period.

Interestingly, a secondary upward trend is observed around the 350th case. This local increase is attributed to multiple complications occurring in a single patient, which temporarily disrupted the overall complication trend despite general surgical proficiency. The significance of the decrease in complications is further emphasized in “Complication rate” section.

### Complication rate

Examining the overall complication rates, a decreasing trend was observed across the patient groups, with 10% complications in the first group (1–100) compared to 8.1% in the second (101–200), 5.1% in the third (201–300), and remaining stable at 5.1% in the fourth group (301–400), before dropping to 1.0% in the last group (401–500) (*p* = 0.008) (Table 2). This confirms a significant time-dependent reduction in complication rate, with the learning curve stabilizing around case 42 according to the CUSUM analysis (Figure 4).

**Table 2:** Postoperative complication rates. TKA = Total Knee Arthroplasty.

TKA (*n*)	1–100	101–200	201–300	301–400	401–481	*p*-value
Postoperative complications % (*n*)	10 (10)	8.08 (8)	5.05 (5)	5.05 (5)	1.01 (1)	0.008*
Patellar resurfacing % (*n*)	2.02 (2)	2.02 (2)	0	0	0	0.08
Patellar luxation % (*n*)	0	4.04 (4)	1.01 (1)	1.01 (1)	0	0.393
Extensor mechanism rupture % (*n*)	1 (1)	0	0	2.02 (2)	0	1
Periprosthetic fracture % (*n*)	1 (1)	0	0	1.01 (1)	0	0.622
Instability % (*n*)	1 (1)	0	1.01 (1)	0	0	0.685
Aseptic loosening % (*n*)	1 (1)	0	0	0	0	0.934
Infection % (*n*)	2.02 (2)	2.02 (2)	3.03 (3)	0	0	0.125
Stiffness % (*n*)	2.02 (2)	0	0	3.03 (3)	1.01 (1)	0.274

Postoperative complication rates across five patient cohorts (each ~ 100 cases). A significant reduction in overall complication rate was observed (from 10% to 1%), suggesting a learning curve effect. Significant values are marked with an asterisk (*). Other specific complications did not reach statistical significance.

Among the specific complications, anterior knee pain led to secondary patellar resurfacing in 2.0% of patients in both the first and second groups, but no such cases were reported in the subsequent groups (*p* = 0.08). Patellar dislocation was observed in 4.0% of cases in the second group, without any difference when compared to subsequent groups (*p* = 0.39). Extensor mechanism rupture and periprosthetic fractures were rare, each occurring in isolated cases (*p* = 1 and *p* = 0.622, respectively).

Infections were recorded at 2.0% in the first two groups and increased to 3.0% in the third, before disappearing in the last two groups (*p* = 0.13). Stiffness followed a similar pattern, peaking at 3.0% in the fourth group (*p* = 0.27).

A Poisson regression was performed to evaluate the evolution of the number of complications across patient groups. The model showed a significant coefficient (β = −0.3848, *p* = 0.008), indicating a significant decrease in the number of complications over successive patient groups. This decrease corresponds to a 31.9% reduction in complication risk from one group to the next.

While some trends suggest a decline in postoperative complications over time, no statistically significant differences were found for most specific complications, except for the overall complication rate (*p* = 0.008).

## Discussion

This study demonstrates that the KNEO^®^ system exhibits a significant learning curve, particularly in terms of operative time, which stabilizes after approximately 150 cases. The reduction in operative time from 83 min for the first cases to 63 min for the last reflects a progressive adaptation to the technical demands of the system. As well, a significant downward trend in complication rates was observed over successive patient groups, suggesting a learning curve effect, with a notable reduction from 10% in the first group to 1% in the last.

The learning curve in TKA has been widely investigated, particularly when introducing new implant designs or surgical technologies. Several studies have attempted to quantify this curve based on the type of prosthesis, the surgical approach, or the surgeon’s level of experience. A comparative summary of the main published studies is provided in Table 3, highlighting the case thresholds at which surgical outcomes tend to stabilize.

**Table 3:** Summary of published studies on the learning curve in total knee arthroplasty. TKA = Total Knee Arthroplasty.

Study	Procedure/Technology	Learning curve threshold	Sample size	Main outcome measure	Key findings
[2]	Various new implant technologies in TKA	~50 cases	Review	Complication rate	Learning curve varies by technology and team experience
[12]	Manual instruments TKA with unrestricted kinematic alignment	~20 cases	60	Operative time	Short learning curve; outcomes comparable to experienced surgeon
[13]	TKA using electronic sensor	~40 cases	164	Operative time	Gradual reduction in time with experience
[14]	Change in implant design in high-volume center	30 cases	*n* = 2,329 (1,061 PFC^®^, 1,268 Genesis™ II)	Operative time, complications	Slight increase in OR time (<5 min); stabilization after 30 cases; no difference in complications
[15]	Robotic-assisted TKA (HURWA)	8–20 cases	50	Operative time, alignment	Improved alignment and OR time after early cases
[16]	Robotic-assisted TKA (NAVIO)	~10 cases	101	Operative time	Quick learning curve, progressive stabilization
[17]	Robotic-assisted TKA	~40 cases	386	Operative time, alignment	Learning curve only for time, not for alignment or gaps
[18]	Team familiarity in TKA	No fixed threshold; progressive gain up to 10 + collaborations	4,276	Operative time	Operative time decreased with prior team collaboration
[19]	Introduction of new TKA implants	15–100 cases	46,363	Revision risk	Higher revision in first cases, stable outcomes after 100
[20]	Anterior approach THA	~50 cases	Registry data	Revision rate	Risk stabilizes after 50 cases
[21]	THA anterior approach (senior vs. junior)	70 cases	547	Operative time, complications	Juniors had higher complication rate; time stabilized after 70 cases
[22]	Robotic-assisted TKA (CORI)	6 cases	500	CUSUM events	CUSUM learning curve short despite early perioperative events
[23]	TKA in hemophilic patients	30 cases	90	Complication rate	Significant decline in complications after 30 cases

The presence of the learning curve based on operative time is consistent with previous findings by Nedopil et al., who reported a similar decline in operative time for a novice surgeon transitioning to manual instruments, with a reduction from 112 min for the first 10 cases to 77 min for the last 10 cases [12]. A study of 4,017 minimally invasive total knee arthroplasties showed a progressive decrease in operative time from 70 to 35 min with the surgeon’s experience [24]. Our results corroborate these data, demonstrating that beyond a certain threshold of procedures, the learning curve reaches a plateau. While Sarpong et al. estimated that fewer than 50 cases are required to achieve proficiency with new implant technology [2], our study suggests a longer learning curve for the KNEO^®^ system. This discrepancy may be attributed to the specific characteristics of the system, such as unique instrumentation or surgical workflow modifications. The findings of Lakra et al., who divided their cohort into groups of 41 cases, also revealed a progressive reduction in operative time, mirroring our observations but over a smaller sample size [13]. Similarly, Moonot et al., analyzing 2,329 TKRs, found that introducing a new implant led to a modest increase in operative time (from 69 to 72.4 min, *p* < 0.05) without significantly affecting hospital stay, early postoperative complications, or revision rates [14]. This aligns with our observations that the initial increase in operative time eventually stabilizes as surgeons gain proficiency. Other technologies might certainly affect the surgical team’s learning curve differently. For instance, Kayani et al. reported a significant inflection point after seven cases in robotic-assisted UKAs, emphasizing that the adaptation period varies depending on technological complexity [25]. Zhang et al. similarly reported that the HURWA Robotic Assisted TKA (RA TKA) system reduced operative time after 8–20 cases (*p* < 0.05) and improved postoperative alignment (77.97% within ± 3° vs. 47.19%, *p* < 0.01) [15]. Bosco et al. [16] conducted a retrospective study on 101 robot-assisted TKA performed with the NAVIO system. The study revealed a significant reduction in operative time after the first 11 cases, suggesting a rapid learning curve. The average operative time was 72.3 min, with a progressive stabilization without compromising alignment accuracy. While these studies have shown short learning curves for the surgeon to adopt the new technology, in the study by Vermue et al. longer learning curves (up to 43 cases) have been seen as well with robot-assisted TKA [17]. These findings have crucial implications for surgical training. Identifying a 150-case learning phase underscores the need for structured support during this period. Supervision through mentorship programs, simulation-based training could potentially accelerate the learning process. Furthermore, the correlation between high surgical volume and improved efficiency, as demonstrated by Maruthappu et al. [18], suggests that surgical experience and team familiarity significantly influence operative performance. Their study reported a reduction in median operative time from 121.9 min for teams with no prior collaborations to 83.4 min for teams with more than 10 shared procedures. This reinforces the value of stable team dynamics in optimizing learning curves and patient outcomes, in addition to individual surgical experience.

The study also identified a notable learning curve regarding postoperative complications, with stabilization occurring after approximately 40 cases. A significant reduction in complication rates was observed beyond this threshold, suggesting that increasing surgeon proficiency and procedural familiarity plays a crucial role in mitigating early surgical risks. This trend aligns with Sarpong et al., who emphasized the impact of case volume on improving postoperative outcomes [2]. Similarly, Peltola et al., analyzing 46,363 knee replacements, demonstrated that certain implant models exhibited a pronounced learning curve, with a notably higher revision risk in the first 15 cases. However, after 100 procedures with the same implant, complication rates significantly decreased, and long-term implant survival became comparable to more established models [19]. Comparisons with other procedures further support these findings. De Steiger et al. observed that in total hip arthroplasty via the direct anterior approach, the learning curve – assessed through cumulative revision rates – stabilized after approximately 50 cases [20]. Foissey et al. analyzed the learning curve in direct anterior approach THA and found that operative time stabilized after 70 cases for senior surgeons and 10 cases for junior surgeons, though complication rates were significantly higher in juniors (12.7% vs. 7.8%, *p* = 0.01) [21]. This highlights the importance of experience in minimizing surgical risks and suggests that structured mentorship programs could help junior surgeons improve outcomes more rapidly. Weaver et al. examined a new RA-TKA system and found an extremely short learning curve (6 cases, CUSUM), though perioperative challenges included 3 excessive bone cuts, 2 soft tissue injuries, and 2 robotic system failures out of 500 cases [22]. Despite these initial difficulties, the absence of severe postoperative complications suggests that technological refinements and training could further optimize results with this approach.

While not comparable to patients with primary osteoarthritis, Vahabi et al. [23] conducted a retrospective study analyzing 90 total knee arthroplasties performed by a single surgeon in hemophilic patients. They found that the rate of perioperative complications significantly decreased, with an improvement observed after the first 30 cases and further optimization after 60 cases. These findings emphasize the importance of adequate training and experience to minimize complications and maximize the long-term success of surgical procedures, highlighting the value of early-phase mentorship and technology-specific expertise.

Our study has several limitations. The sample size, while substantial, is limited to two surgical centers, which may affect the generalizability of our findings. Second, our analysis did not account for potential confounding factors such as patient comorbidities. Third, the retrospective nature of the study introduces inherent biases, including potential underreporting of minor complications. Finally, structured training programs incorporating simulation and mentorship could be explored to accelerate proficiency while minimizing early complications, as suggested by previous studies on surgical training and learning curves [21–23].

## Conclusion

This study demonstrates that the adoption of the KNEO^®^ total knee arthroplasty system follows a significant learning curve, with operative times stabilizing after approximately 150 procedures. A progressive reduction in surgical duration was observed, with a total decrease of 16.7%, reflecting improved efficiency and familiarity with the system. More importantly, the analysis of postoperative complications revealed a 32% reduction in complication risk per group of consecutive 100 patients, highlighting the direct impact of surgical experience on patient safety and outcomes. These findings underscore the importance of structured training, and gradual case exposure in optimizing the learning curve for novel implant systems.

## Data Availability

The datasets analyzed in this study are not publicly available due to institutional data privacy policies, but are available from the corresponding author on reasonable request.

## References

[1] Markos VE, Paul WM, Christina K (2019) Learning curve analysis of minimally invasive total knee arthroplasty using segmented linear regression modelling. J Chin Med Assoc 82(11), 883–887.31135573 10.1097/JCMA.0000000000000128

[2] Sarpong NO, Herndon CL, Held MB, Neuwirth AL, Hickernell TR, Geller JA, Shah RP (2020) What is the learning curve for new technologies in total joint arthroplasty? A review, Curr Rev Musculoskelet Med 13(6), 675–684. 32827304 10.1007/s12178-020-09671-7 PMC7661627

[3] Kashyap S, van Ommeren JW, Shankar S (2008) Minimally invasive surgical technique in total knee arthroplasty: A learning curve. Surg Innov 16(1), 55–59. 10.1177/1553350609331396 19287001

[4] Acuña AJ, Samuel LT, Karnuta JM, Sultan AA, Swiergosz A, Kamath AF (2019) What factors influence operative time in total knee arthroplasty? A 10-year analysis in a national sample. J Arthroplasty 35(3), 621–627. 31767239 10.1016/j.arth.2019.10.054

[5] Lei T, Jiang Z, Qian H, Backstein D, Lei P, Hu Y (2022) Comparison of single-radius with multiple-radius femur in total knee arthroplasty: a meta-analysis of prospective randomized controlled trials. Orthop Surg 14(10), 2085–2093. 35924690 10.1111/os.13391 PMC9483041

[6] Indelli PF (2015) Modern total knee arthroplasty designs: Are we improving outcomes? MOJ Orthop Rheumatol 3(3), 00120.

[7] Nisar S, Palan J, Rivière C, Emerton M, Pandit H (2020) Kinematic alignment in total knee arthroplasty. EFORT Open Rev 5(6), 380–389. 32818065 10.1302/2058-5241.5.200010 PMC7407864

[8] Lee Y, Ha Y, Hwang DS, Koo K (2013) Learning curve of basic hip arthroscopy technique: CUSUM analysis. Knee Surg Sports Traumatol Arthrosc 21(8), 1940–1946. 23073816 10.1007/s00167-012-2241-x

[9] Flevas DA, Megaloikonomos PD, Dimopoulos L, Mitsiokapa E, Koulouvaris P, Mavrogenis AF (2018) Thromboembolism prophylaxis in orthopaedics: an update. EFORT Open Rev 3(4), 136–148. 29780621 10.1302/2058-5241.3.170018 PMC5941651

[10] Blakeney WG, Vendittoli PA. 2020. Restricted kinematic alignment: the ideal compromise. In: Personalized hip and knee joint replacement. Rivière C, Vendittoli PA, Editors. Cham, CH, Springer. 33347126

[11] Yap C, Colson M, Watters D (2007) Cumulative sum techniques for surgeons: A brief review. ANZ J Surg 77(7), 583–586. 17610698 10.1111/j.1445-2197.2007.04155.x

[12] Nedopil AJ, Dhaliwal A, Howell SM, Hull ML (2022) A surgeon that switched to unrestricted kinematic alignment with manual instruments has a short learning curve and comparable resection accuracy and outcomes to those of an experienced surgeon. J Pers Med 12(8), 1152. 35887649 10.3390/jpm12071152 PMC9320158

[13] Lakra A, Sarpong NO, Jennings E, Grosso MJ, Cooper HJ, Shah RP, Geller JA (2019) The learning curve by operative time for soft tissue balancing in total knee arthroplasty using electronic sensor technology. J Arthroplasty 34(3), 483–488. 30528677 10.1016/j.arth.2018.11.014

[14] Moonot P, D’Mello O, Tzinga N, Sisák K, Fiddian N, Harvey A (2013) Impact of change of knee prosthesis on early clinical outcomes in a large volume arthroplasty centre. Ann R Coll Surg Engl 95(8), 573–576. 24165339 10.1308/003588413X13629960046796 PMC4311533

[15] Zhang H, Bai X, Wang H, Zhu Z, Li X (2023) Learning curve analysis of robotic-assisted total knee arthroplasty with a Chinese surgical system. J Orthop Surg Res 18, 843. 38012732 10.1186/s13018-023-04382-4 PMC10680304

[16] Bosco F, Rovere G, Burgio C, Bue GL, Cobisi CD, Via RG, Lucenti L, Camarda L (2025) Accuracy and learning curve of imageless robotic-assisted total knee arthroplasty. J Orthop 66, 77–83. 39896862 10.1016/j.jor.2024.12.029 PMC11779652

[17] Vermue H, Luyckx T, de Grave PW, Ryckaert A, Cools AS, Himpe N, Victor J (2022) Robot-assisted total knee arthroplasty is associated with a learning curve for surgical time but not for component alignment, limb alignment and gap balancing. Knee Surg Sports Traumatol Arthrosc 30(2), 593–601. 33141246 10.1007/s00167-020-06341-6

[18] Maruthappu M, Duclos A, Zhou C, Lipsitz SR, Wright J, Orgill DP, Carty MJ (2016) The impact of team familiarity and surgical experience on operative efficiency: a retrospective analysis. J R Soc Med 109(4), 147–153. 27053357 10.1177/0141076816634317 PMC4827107

[19] Peltola M, Malmivaara A, Paavola M (2013) Learning curve for new technology? Nationwide register-based study of 46, 363 total knee arthroplasties. J Bone Joint Surg Am 95(24), 2097–2103. 24306696 10.2106/JBJS.L.01296

[20] de Steiger RN, Lorimer M, Solomon M (2015) What is the learning curve for the anterior approach for total hip arthroplasty? Clin Orthop Relat Res 473(12), 3860–3866. 26394641 10.1007/s11999-015-4565-6 PMC4626490

[21] Foissey C, Fauvernier M, Fary C, Servien E, Lustig S, Batailler C (2020) Total hip arthroplasty performed by direct anterior approach–Does experience influence the learning curve? SICOT J 6, 15. 32500856 10.1051/sicotj/2020015 PMC7273835

[22] Weaver DJ, Deshmukh S, Bashyal RK, Bagaria V (2024) Complications and learning curve associated with an imageless burr-based (CORI) robotic-assisted total knee arthroplasty system: Results from first 500 cases. Indian J Orthop 58(6), 1109–1115. 39087033 10.1007/s43465-024-01200-9 PMC11286604

[23] Vahabi A, Biçer EK, Aydoğdu S (2024) Total knee arthroplasty in hemophilic knees requires its own learning phase: lessons learned from 90 cases. Knee 53, 28–34. 39667101 10.1016/j.knee.2024.11.024

[24] Cheng YC, Wu P, Chen C, Chen CM, Tsai S, Chang MC, Chen W (2019) Analysis of learning curve of minimally invasive total knee arthroplasty. J Chin Med Assoc 82(7), 576–582. 31021883 10.1097/JCMA.0000000000000118 PMC13048205

[25] Kayani B, Konan S, Pietrzak JRT, Huq SS, Tahmassebi J, Haddad FS (2018) The learning curve associated with robotic-arm assisted unicompartmental knee arthroplasty. Bone Joint J 100(8), 1033–1042. 30062950 10.1302/0301-620X.100B8.BJJ-2018-0040.R1

